# Update: Recommendations of the Advisory Committee on Immunization Practices for Use of Hepatitis A Vaccine for Postexposure Prophylaxis and for Preexposure Prophylaxis for International Travel

**DOI:** 10.15585/mmwr.mm6743a5

**Published:** 2018-11-02

**Authors:** Noele P. Nelson, Ruth Link-Gelles, Megan G. Hofmeister, José R. Romero, Kelly L. Moore, John W. Ward, Sarah F. Schillie

**Affiliations:** ^1^Division of Viral Hepatitis, National Center for HIV/AIDS, Viral Hepatitis, STD, and TB Prevention, CDC; ^2^Pediatric Infectious Diseases Section, University of Arkansas for Medical Sciences and Arkansas Children’s Hospital, Arkansas Children’s Hospital Research Institute, Little Rock, Arkansas; ^3^Division of Communicable and Environmental Diseases and Emergency Preparedness, Tennessee Department of Health.

Postexposure prophylaxis (PEP) with hepatitis A (HepA) vaccine or immune globulin (IG) effectively prevents infection with hepatitis A virus (HAV) when administered within 2 weeks of exposure. Preexposure prophylaxis against HAV infection through the administration of HepA vaccine or IG provides protection for unvaccinated persons traveling to or working in countries that have high or intermediate HAV endemicity. The Advisory Committee on Immunization Practices (ACIP) Hepatitis Vaccines Work Group conducted a systematic review of the evidence for administering vaccine for PEP to persons aged >40 years and reviewed the HepA vaccine efficacy and safety in infants and the benefits of protection against HAV before international travel. The February 21, 2018, ACIP recommendations update and supersede previous ACIP recommendations for HepA vaccine for PEP and for international travel. Current recommendations include that HepA vaccine should be administered to all persons aged ≥12 months for PEP. In addition to HepA vaccine, IG may be administered to persons aged >40 years depending on the provider’s risk assessment. ACIP also recommended that HepA vaccine be administered to infants aged 6–11 months traveling outside the United States when protection against HAV is recommended. The travel-related dose for infants aged 6–11 months should not be counted toward the routine 2-dose series. The dosage of IG has been updated where applicable (0.1 mL/kg). HepA vaccine for PEP provides advantages over IG, including induction of active immunity, longer duration of protection, ease of administration, and greater acceptability and availability.

## Introduction

Postexposure prophylaxis (PEP) with hepatitis A (HepA) vaccine or immune globulin (IG) effectively prevents infection with hepatitis A virus (HAV) when administered within 2 weeks of exposure (*1*,*2*). The efficacy of IG or vaccine when administered >2 weeks after exposure has not been established. Previous ACIP* recommendations for PEP included HepA vaccine for persons aged 1–40 years and IG for persons outside this age range; if IG was not available for persons aged >40 years, HepA vaccine could be administered (*1*).

Preexposure prophylaxis against HAV infection through the administration of HepA vaccine or IG is also recommended for unvaccinated persons traveling to or working in countries that have high or intermediate HAV endemicity (*3*). Because HepA vaccine is not licensed for use in children aged <1 year, IG has historically been recommended for travelers in this age group; however, IG cannot be administered simultaneously with measles, mumps, and rubella (MMR) vaccine, which is also recommended for infants aged 6–11 months traveling internationally from the United States (*4*–*6*).

This report provides recommendations for PEP use of HepA vaccine and IG, and use of HepA vaccine and IG for preexposure protection for persons who will be traveling internationally, including infants aged 6–11 months. This report updates and supersedes previous ACIP recommendations for HepA vaccine for PEP and for international travel (*1*).

## Methods

During November 2016–February 2018, the ACIP Hepatitis Work Group^†^ held monthly conference calls to review and discuss relevant scientific evidence,^§^ including the use of HepA vaccine and IG for PEP and the use of HepA vaccine for infants before some international travel. The ACIP Hepatitis Work Group evaluated the quality of evidence related to the benefits and harms of administering a dose of HepA vaccine for PEP for persons aged >40 years using the Grading of Recommendations Assessment, Development, and Evaluation (GRADE) framework (https://www.cdc.gov/vaccines/acip/recs/grade/table-refs.html). Quality of evidence related to the benefits and harms of administering HepA vaccine for preexposure prophylaxis to infants aged 6–11 months who will be traveling internationally was not evaluated using the GRADE framework; instead, studies of HepA vaccine efficacy and safety in infants (*7*–*9*) and the benefits of protection against HAV before international travel were considered (*3*).

At the February 2018 ACIP meeting, the following proposed recommendations were presented to the committee: 1) HepA vaccines should be administered for PEP for all persons aged ≥12 months; in addition to HepA vaccine, IG may be administered to persons aged >40 years for PEP, depending on the provider’s risk assessment; and 2) HepA vaccine should be administered to infants aged 6–11 months traveling outside the United States when protection against hepatitis A is recommended. After a period for public comment, the recommendations were approved unanimously by the voting ACIP members.^¶^

## Summary of Key Findings

**Prevention of HAV infection with HepA vaccine following exposure.** A randomized, double-blind clinical trial of HepA vaccine in 1,090 HAV-susceptible persons aged 2–40 years who were contacts of persons with HAV infection suggested that performance of HepA vaccine administered <14 days after exposure approaches that of IG in healthy children and adults aged <40 years (*1*,*10*). Limited data are available comparing HepA vaccine and IG in healthy adults aged >40 years; available data indicate reduced response to HepA vaccine in older age groups compared with response in younger adults (*11*).

**GRADE quality of evidence summary for HepA vaccine for PEP in persons aged >40 years.** The evidence assessing benefits and harms of administering a dose of HepA vaccine for PEP to prevent HAV infection in adults aged >40 years was determined to be GRADE evidence type 4 (i.e., evidence from clinical experience and observations, observational studies with important limitations, or randomized controlled trials with several major limitations) for benefits and type 3 (i.e., evidence from observational studies, or randomized controlled trials with notable limitations) for harms (https://www.cdc.gov/vaccines/acip/recs/grade/table-refs.html).

**Prevention of HAV infection among infants aged 6–11 months who received HepA vaccine before travel.** HepA vaccine was demonstrated to be safe and efficacious for infants as young as age 2 months (*2*,*7*–*9*), although vaccination of infants aged <12 months might result in a suboptimal immune response because of potential interference with passively acquired maternal antibody, which could decrease long-term immunity (*7*–*9*).

## Rationale for Recommendations

**Advantages of HepA vaccine for PEP.** HepA vaccine for PEP provides numerous public health advantages compared with IG, including the induction of active immunity and longer duration of protection, ease of administration, and greater acceptability and availability (*11*). Previous recommendations favoring IG for adults aged >40 years were based on the premise that IG is more efficacious in this group; however, evidence of decreased IG potency (i.e., reduced titers of anti-HAV antibodies) (*12*) led to a recommendation for an increase in the IG dosage (0.1 mL/kg) for hepatitis A PEP in 2017, with a consequent increase in IG administration volume (*6*). In addition, when HAV exposure, and thus the need for PEP, is not clear (i.e., consumer of recalled food product or patron at a restaurant where a notification occurred), the benefit of IG compared with vaccine, which provides long-term protection, is less certain.

**Before travel administration of HepA vaccine to infants aged 6–11 months.** IG cannot be administered simultaneously with MMR vaccine because antibody-containing products such as IG can inhibit the immune response to measles and rubella vaccines for 3 months (*4*,*6*). However, because MMR vaccine is recommended for all infants aged 6–11 months traveling internationally from the United States and because measles in infancy is more severe than HAV infection in infancy, MMR vaccine should be administered preferentially to preexposure prophylaxis with IG for prevention of HAV infection. Administration of HepA vaccine (indication for off-label use) and MMR vaccine to infants aged 6–11 months (*7*–*9*) provides protection against both HAV and measles and allows for simultaneous prophylactic administration (*4*,*13*).

## Recommendations for Postexposure Prophylaxis Against HAV Infection

HepA vaccine should be administered to all persons aged ≥12 months for PEP. In addition to HepA vaccine, IG may be administered to persons aged >40 years, depending on the provider’s risk assessment (Supplementary Text 1, https://staging-stacks.cdc.gov/view/cdc/59777). Recommendations for PEP have been updated to include HepA vaccine for all unvaccinated persons aged ≥12 months, regardless of risk group, and co-administration of IG when indicated (Table 1). The dosage of GamaSTAN S/D human IG for PEP (0.1 mL/kg) also has been updated (*6*). Persons who have recently been exposed to HAV and who have not received HepA vaccine previously should receive PEP as soon as possible, within 2 weeks of exposure (*1*).

**TABLE 1:** Recommendations for postexposure prophylaxis and preexposure protection, by age group and risk category

Indication/Age group	Risk category/Health status	Hepatitis A vaccine	Immune globulin
**Postexposure prophylaxis**			
<12 mos	Healthy	No	0.1 mL/kg*
12 mos–40 yrs	Healthy	1 dose^†^	None
>40 yrs	Healthy	1 dose^†^	0.1 mL/kg^§^
≥12 mos	Immunocompromised or chronic liver disease	1 dose^†^	0.1 mL/kg^¶^
≥12 mos	Vaccine contraindicated**	No	0.1 mL/kg
**Preexposure protection^††^**			
<6 mos	Healthy	No	0.1–0.2 mL/kg^§§^
6–11 mos	Healthy	1 dose^¶¶^	None
12 mos–40 yrs	Healthy	1 dose***	None
>40 yrs	Healthy	1 dose***	0.1–0.2 mL/kg^§§,†††^
All ages	Immunocompromised or chronic liver disease	1 dose***	0.1–0.2 mL/kg^§§,†††^
>6 mos	Persons who elect not to receive vaccine or for whom vaccine is contraindicated**	No	0.1–0.2 mL/kg^§§^

* Measles, mumps, and rubella vaccine should not be administered for at least 3 months after receipt of IG.

^†^ A second dose is not required for postexposure prophylaxis; however, for long-term immunity, the hepatitis A vaccination series should be completed with a second dose at least 6 months after the first dose.

^§^ The provider’s risk assessment should determine the need for immune globulin administration. If the provider’s risk assessment determines that both vaccine and immune globulin are warranted, HepA vaccine and immune globulin should be administered simultaneously at different anatomic sites

^¶^ Vaccine and immune globulin should be administered simultaneously at different anatomic sites.

** Life-threatening allergic reaction to a previous dose of hepatitis A vaccine, or allergy to any vaccine component.

^††^ IG should be considered before travel for persons with special risk factors for either HAV infection or increased risk for complications in the event of exposure to HAV.

^§§^ 0.1 mL/kg for travel up to 1 month; 0.2 mL/kg for travel up to 2 months, 0.2mL/kg every 2 months for travel of ≥2 months’ duration.

^¶¶^ This dose should not be counted toward the routine 2-dose series, which should be initiated at age 12 months.

*** For persons not previously vaccinated with HepA vaccine, administer dose as soon as travel is considered, and complete series according to routine schedule.

^†††^ May be administered, based on providers’ risk assessment.

**Infants aged <12 months and persons for whom vaccine is contraindicated**. Infants aged <12 months and persons for whom vaccine is contraindicated (persons who have had a life-threatening allergic reaction after a dose of HepA vaccine, or who have a severe allergy to any component of this vaccine) should receive IG (0.1 mL/kg) (*6*,*14*) instead of HepA vaccine, as soon as possible and within 2 weeks of exposure. MMR and varicella vaccines should not be administered sooner than 3 months after IG administration (*4*–*6*).

**Immunocompetent persons aged ≥12 months**. Persons aged ≥12 months who have been exposed to HAV within the past 14 days and have not previously completed the 2-dose HepA vaccine series should receive a single dose of HepA vaccine (Table 2) as soon as possible. In addition to HepA vaccine, IG (0.1 mL/kg) may be administered to persons aged >40 years depending on the providers’ risk assessment (Supplementary Text 1, https://staging-stacks.cdc.gov/view/cdc/59777). For long-term immunity, the HepA vaccine series should be completed with a second dose at least 6 months after the first dose; however, the second dose is not necessary for PEP. A second dose should not be administered any sooner than 6 months after the first dose, regardless of HAV exposure risk.

**TABLE 2:** Vaccines used to prevent hepatitis A virus (HAV) infection

Vaccine	Trade name (manufacturer)	Age group (yrs)	Dosage	Route	Schedule	Booster
Hepatitis A vaccine, inactivated	Havrix (GlaxoSmithKline)	1–18	0.5 mL (720 ELU)	IM	0, 6–12 mo	None
		≥19	1 mL (1,440 ELU)	IM	0, 6–12 mo	None
Hepatitis A vaccine, inactivated	Vaqta (Merck and Co.)	1–18	0.5 mL (25 U)	IM	0, 6–18 mo	None
		≥19	1 mL (50 U)	IM	0, 6–18 mo	None
Combined hepatitis A and B vaccine*	Twinrix (GlaxoSmithKline)	≥18 (primary)	1 mL (720 ELU HAV + 20 *μ*g HBsAg)	IM	0, 1, 6 mo	None
		≥18 (accelerated)	1 mL (720 ELU HAV + 20 *μ*g HBsAg)	IM	0, 7, 21–30 days	12 mo

**Abbreviations:** ELU = ELISA units of inactivated HAV; HBsAg = hepatitis B surface antigen; IM = intramuscular; U = units of HAV antigen.

* Combined hepatitis A and B vaccine (Twinrix) should not be used for postexposure prophylaxis.

**Persons aged ≥12 months who are immunocompromised or have chronic liver disease.** Persons who are immunocompromised or have chronic liver disease and who have been exposed to HAV within the past 14 days and have not previously completed the 2-dose HepA vaccination series should receive both IG (0.1 mL/kg) and HepA vaccine simultaneously in a different anatomic site (e.g., separate limbs) as soon as possible after exposure (*6*,*15*–*17*) (Table 1). For long-term immunity, the HepA vaccination series should be completed with a second dose at least 6 months after the first dose; however, the second dose is not necessary for PEP. A second dose should not be administered any sooner than 6 months after the first dose, regardless of HAV exposure risk.

In addition to HepA vaccine, IG should be considered for postexposure prophylaxis for persons with special risk factors for either HAV infection or increased risk of complications in the event of an exposure to HAV (Table 3) (Supplementary Text 1, https://staging-stacks.cdc.gov/view/cdc/59777).

**TABLE 3:** Categories of persons with increased risk for hepatitis A virus (HAV) infection or increased risk for complications in the event of exposure to HAV

Type of risk	Risk category	Examples
**Increased risk for HAV infection**	Close contacts of persons with HAV infection*	Household contacts
		Caretakers
		Sexual contacts
	Occupational risk	Persons working with nonhuman primates
		Persons working with HAV in a research laboratory
**Increased risk for HAV-associated complications**	Immunocompromised persons	Congenital or acquired immunodeficiency
		HIV infection
		Chronic renal failure/Undergoing dialysis
		Solid organ, bone marrow, or stem cell transplant recipients
		Persons with diseases requiring treatment with immunosuppressive drugs/biologics (e.g., tumor necrosis alpha inhibitors), long-term systemic corticosteroids, radiation therapy
	Chronic liver disease	Hepatitis B infection
		Hepatitis C infection
		Cirrhosis (any etiology)
		Fatty liver disease (hepatic steatosis)
		Alcoholic liver disease
		Autoimmune hepatitis
		Alanine aminotransferase (ALT) or aspartate amino transferase (AST) level more than twice the upper limit of normal or persistently elevated for 6 months

**Abbreviation:** HIV = human immunodeficiency virus.

* Excludes health care personnel using appropriate personal protective equipment.

## Recommendations for Preexposure Protection Against HAV Infection for Travelers

**Infants aged 6–11 months.** HepA vaccine should be administered to infants aged 6–11 months traveling outside the United States when protection against HAV is recommended (Table 1). The travel-related dose for infants aged 6–11 months should not be counted toward the routine 2-dose series. Therefore, the 2-dose HepA vaccination series should be initiated at age 12 months according to the routine, age-appropriate vaccination schedule.

Recommendations for preexposure protection against HAV for travelers aged <6 months and aged ≥12 months remain unchanged from previous recommendations (Table 1), except for the updated dosage of IG where applicable (Supplementary Text 2, https://staging-stacks.cdc.gov/view/cdc/59778) (*6*). For travel duration up to 1 month, 0.1 mL/kg of IG is recommended; for travel up to 2 months, the dose is 0.2 mL/kg, and for travel of ≥2 months, a 0.2 mL/kg dose should be repeated every 2 months for the duration of travel. All susceptible persons traveling to or working in countries that have high or intermediate HAV endemicity are at increased risk for infection and should be vaccinated or receive IG before departure (*1*,*3*).

**Infants aged <6 months and travelers who elect not to receive vaccine or for whom vaccine is contraindicated.** Infants aged <6 months and travelers who elect not to receive vaccine or for whom vaccine is contraindicated should receive a single dose of IG before travel when protection against HAV is recommended. If travel is for ≥2 months’ duration, a repeat dose of 0.2 mL/kg every 2 months should be administered (*6*).

**Healthy persons aged 12 months–40 years.** Healthy persons aged 12 months–40 years who are planning travel to an area with high or intermediate HAV endemicity and have not received HepA vaccine should receive a single dose of HepA vaccine as soon as travel is considered and should complete the 2-does series according to the routine schedule.

**Persons aged >40 years, immunocompromised persons, and persons with chronic liver disease.** Persons with chronic liver disease as well as adults aged >40 years, immunocompromised persons, and persons with other chronic medical conditions planning to depart to an area with high or intermediate HAV endemicity in <2 weeks should receive the initial dose of HepA vaccine, and also simultaneously may be administered IG at a separate anatomic injection site (e.g., separate limbs) (Table 1) (*6*,*15*–*17*).

In addition to HepA vaccine, IG should be considered before travel for persons with special risk factors for either HAV infection or increased risk for complications in the event of an exposure to HAV (Table 3) (Supplementary Text 2, https://staging-stacks.cdc.gov/view/cdc/59778).

**Summary**

**What is already known about this topic?**

Postexposure prophylaxis (PEP) with hepatitis A (HepA) vaccine or immune globulin (IG) prevents infection with hepatitis A virus when administered within 2 weeks of exposure. Measles, mumps, and rubella vaccine (MMR) is recommended for infants aged 6–11 months traveling outside the United States. IG cannot be administered simultaneously with MMR.

**What is added by this report?**

HepA vaccine is recommended for persons aged ≥12 months for PEP. Providers may also administer IG to adults aged >40 years, if indicated. The dosage of IG has been updated. Simultaneous administration of MMR and HepA vaccines is recommended for infants aged 6–11 months traveling internationally.

**What are the implications for public health practice?**

HepA vaccine for PEP provides advantages over IG, including induction of active immunity, longer duration of protection, ease of administration, and greater acceptability and availability.

## Footnotes

* Recommendations for routine use of vaccines in children, adolescents, and adults are developed by the Advisory Committee on Immunization Practices (ACIP). ACIP is chartered as a federal advisory committee to provide expert external advice and guidance to the Director of CDC on use of vaccines and related agents for the control of vaccine-preventable diseases in the civilian U.S. population. Recommendations for routine use of vaccines in children and adolescents are harmonized to the greatest extent possible with recommendations made by the American Academy of Pediatrics (AAP), the American Academy of Family Physicians (AAFP), and the American College of Obstetricians and Gynecologists (ACOG). Recommendations for routine use of vaccines in adults are harmonized with the recommendations of AAFP, ACOG, and the American College of Physicians (ACP). ACIP recommendations approved by the CDC Director become agency guidelines on the date published in the Morbidity and Mortality Weekly Report (MMWR). https://www.cdc.gov/vaccines/acip.

† The ACIP Hepatitis Vaccines Work Group comprises professionals from academic medicine (family medicine, internal medicine, pediatrics, obstetrics, infectious disease, occupational health, and preventive medicine specialists), federal and state public health entities, and medical societies.

§ In preparation for ACIP deliberation, the scientific literature was searched using PubMed and EMBASE databases for reports published from January 1, 1992, through January 7, 2017. Search terms included “hepatitis A vaccine” and “HAV vaccine” and excluded studies in nonhumans and articles on children and adolescents. To qualify as a candidate for inclusion in the review, a study had to include data within 2 weeks of the first dose of HepA vaccine. Studies were excluded if they reported data focused solely on children, did not provide information on ages of persons studied, did not include data on Havrix or Vaqta (the two single antigen HepA vaccines currently licensed in the United States), only included safety data or discussed vaccine introduction without providing new data on vaccine efficacy or seroprotection, or only reported data on persons with underlying health conditions.

¶ 14 voted in favor, with none opposed, none abstained, and none recused.
