# *Erratum:* Vol. 67, No. 43

**DOI:** 10.15585/mmwr.mm6809a4

**Published:** 2019-03-08

In the report “Update: Recommendations of the Advisory Committee on Immunization Practices for Use of Hepatitis A Vaccine for Postexposure Prophylaxis and for Preexposure Prophylaxis for International Travel,” errors occurred in Table 1. The corrected Table 1 is below.

**TABLE 1:** Recommendations for postexposure prophylaxis and preexposure protection, by age group and risk category

Indication/Age group	Risk category/Health status	Hepatitis A vaccine	Immune globulin*
**Postexposure prophylaxis**			
<12 mos	Healthy	No	0.1 mL/**kg**
12 mos–40 yrs	Healthy	1 dose^†^	None
>40 yrs	Healthy	1 dose^†^	0.1 mL/kg^§^
≥12 mos	Immunocompromised or chronic liver disease	1 dose^†^	0.1 mL/kg^¶^
≥12 mos	Vaccine contraindicated**	No	0.1 mL/kg
**Preexposure protection^††^**			
<6 mos	Healthy	No	0.1–0.2 mL/kg^§§^
6–11 mos	Healthy	1 dose^¶¶^	None
12 mos–40 yrs	Healthy	1 dose***	None
>40 yrs	Healthy	1 dose***	0.1–0.2 mL/kg^§§,†††^
**>6 mos**	Immunocompromised or chronic liver disease	1 dose***	0.1–0.2 mL/kg^§§,†††^
>6 mos	Persons who elect not to receive vaccine or for whom vaccine is contraindicated	No	0.1–0.2 mL/kg^§§^

* Measles, mumps, and rubella vaccine should not be administered for at least 3 months after receipt of immune globulin.

^†^ A second dose is not required for postexposure prophylaxis; however, for long-term immunity, the hepatitis A vaccination series should be completed with a second dose at least 6 months after the first dose.

^§^ The provider’s risk assessment should determine the need for immune globulin administration. If the provider’s risk assessment determines that both vaccine and immune globulin are warranted, Hepatitis A vaccine and immune globulin should be administered simultaneously at different anatomic sites.

^¶^ Vaccine and immune globulin should be administered simultaneously at different anatomic sites.

** Life-threatening allergic reaction to a previous dose of hepatitis A vaccine, or allergy to any vaccine component.

^††^ Immune globulin should be considered before travel for persons with special risk factors for either hepatitis A virus (HAV) infection or increased risk for complications in the event of exposure to HAV.

^§§^ 0.1 mL/kg for travel up to 1 month; 0.2 mL/kg for travel up to 2 months, 0.2 mL/kg every 2 months for travel of ≥2 months’ duration.

^¶¶^ This dose should not be counted toward the routine 2-dose series, which should be initiated at age 12 months.

*** For persons not previously vaccinated with HepA vaccine, administer dose as soon as travel is considered, and complete series according to routine schedule.

^†††^ May be administered, based on providers’ risk assessment.

